# Analysis of genomic and non-genomic signaling of estrogen receptor in PDX models of breast cancer treated with a combination of the PI3K inhibitor alpelisib (BYL719) and fulvestrant

**DOI:** 10.1186/s13058-021-01433-8

**Published:** 2021-05-21

**Authors:** Julien Jacquemetton, Loay Kassem, Coralie Poulard, Ahmed Dahmani, Ludmilla De Plater, Elodie Montaudon, Laura Sourd, Ludivine Morisset, Rania El Botty, Sophie Chateau-Joubert, Sophie Vacher, Ivan Biche, Isabelle Treilleux, Olivier Trdan, Elisabetta Marangoni, Muriel Le Romancer

**Affiliations:** 1 Universit de Lyon, F-69000 Lyon, France; 2 Inserm U1052, Centre de Recherche en Cancrologie de Lyon, F-69000 Lyon, France; 3 CNRS UMR5286, Centre de Recherche en Cancrologie de Lyon, F-69000 Lyon, France; 4 Clinical Oncology Department, Faculty of Medicine, Cairo University, Cairo, Egypt; 5 Translational Research Department, Institut Curie, PSL University, 75005 Paris, France; 6 cole Nationale Vtrinaire dAlfort, BioPle Alfort, 94704 Maisons-Alfort Cedex, France; 7 Genetics Department, Institut Curie, Paris, France; 8 Pathology Department, Centre Lon Brard, F-69000 Lyon, France; 9 Medical Oncology Department, Centre Lon Brard, F-69000 Lyon, France; 10 Centre de Recherche en Cancrologie de Lyon, INSERM 1052, CNRS 5286, Centre Lon Brard, Btiment D, 28 rue Laennec, 69373 Lyon Cedex 08, France

**Keywords:** Breast cancer, Estrogen signaling, Resistance, PI3K, PDX, Biomarker

## Abstract

**Background:**

Endocrine therapies targeting estrogen signaling have significantly improved breast cancer (BC) patient survival, although 40% of ER-positive BCs do not respond to those therapies. Aside from genomic signaling, estrogen triggers non-genomic pathways by forming a complex containing methylER/Src/PI3K, a hallmark of aggressiveness and resistance to tamoxifen. We aimed to confirm the prognostic value of this complex and investigated whether its targeting could improve tumor response in vivo.

**Methods:**

The interaction of ER/Src and ER/PI3K was studied by proximity ligation assay (PLA) in a cohort of 440 BC patients. We then treated patient-derived BC xenografts (PDXs) with fulvestrant or the PI3K inhibitor alpelisib (BYL719) alone or in combination. We analyzed their anti-proliferative effects on 6 ER+ and 3 ER PDX models. Genomic and non-genomic estrogen signaling were assessed by measuring ER/PI3K interaction by PLA and the expression of estrogen target genes by RT-QPCR, respectively.

**Results:**

We confirmed that ER/Src and ER/PI3K interactions were associated with a trend to poorer survival, the latter displaying the most significant effects. In ER+ tumors, the combination of BYL719 and fulvestrant was more effective than fulvestrant alone in 3 models, irrespective of PI3K, PTEN status, or ER/PI3K targeting. Remarkably, resistance to fulvestrant was associated with non-genomic ER signaling, since genomic degradation of ER was unaltered in these tumors, whereas the treatment did not diminish the level of ER/PI3K interaction. Interestingly, in 2 ER models, fulvestrant alone impacted tumor growth, and this was associated with a decrease in ER/PI3K interaction.

**Conclusions:**

Our results demonstrate that ER/PI3K may constitute a new prognostic marker, as well as a new target in BC. Indeed, resistance to fulvestrant in ER+ tumors was associated with a lack of impairment of ER/PI3K interaction in the cytoplasm. In addition, an efficient targeting of ER/PI3K in ER tumors could constitute a promising therapeutic option.

**Supplementary Information:**

The online version contains supplementary material available at 10.1186/s13058-021-01433-8.

## Background

Breast cancer (BC) is the most common cancer among women worldwide [1]. More than 75% of breast tumors express the estrogen receptor (ER in the nucleus and are commonly categorized as luminal BCs). ER plays a major role in BC tumorigenesis as it regulates cell cycle, cell survival, and angiogenesis [2]. Interfering with the ER pathway using anti-estrogens (selective estrogen receptor modulators such as tamoxifen or selective estrogen downregulators such as fulvestrant) or through estrogen deprivation (e.g., aromatase inhibitors) increases the survival of ER-positive BC patients. Despite the high level of sensitivity of luminal tumors to endocrine therapy, treatment efficacy is limited by intrinsic and acquired resistance [3, 4]. Indeed, 3050% of patients relapse after adjuvant treatment and eventually die from metastases [5].

The *PIK3CA gene*, encoding the p110 subunit of PI3K, is mutated in 4050% of ER+ tumors, suggesting a dependency of ER+ breast cancer cells on this pathway [6, 7]. Given the role of PI3K in supporting proliferation, survival, and hormone receptor pathway activity, it is not surprising that activation of the PI3K/AKT/mTOR pathway promotes disease progression and resistance to endocrine therapy [8]. *PIK3CA*-mutated preclinical cancer models are sensitive to PI3K inhibitors, which appear to function synergistically with endocrine therapies [9]. This was recently confirmed in patients, as treatment with alpelisib (PI3K inhibitor) combined to fulvestrant prolonged survival of *PIK3CA*-mutated patients [10]. At the molecular level, the ER and PI3K pathways crosstalk at different levels [3]. At the genomic level, somatic activating mutations of the *PIK3CA* gene lead to abnormal PI3K/AKT/mTOR pathway activation [11]. In addition, PI3K inhibition increases ER transcriptional activity via SGK1 and a feedback mechanism that attenuates the activity of PI3K inhibitors [12]. Beyond these genomic mechanisms of action, activation of the PI3K pathway in BC can occur via a non-genomic signaling pathway involving cytoplasmic ER [13, 14]. Cytoplasmic ER when complexed to Src and PI3K activates Akt, triggering proliferation and cell survival [13, 15,17]. Our team reported that methylation of ER on residue R260 by the arginine methyltransferase PRMT1 is a prerequisite for its association with Src and PI3K and the activation of Akt [18, 19]. Subsequently, using the proximity ligation assay (PLA) methodology to detect in situ protein/protein interactions [20], we showed that this pathway, characterized by the formation of ER/Src/PI3K, is present in normal breast tissue and is hyperactivated in aggressive breast tumors [21]. Moreover, we unveiled that ER/Src and ER/PI3K interactions are associated with resistance to tamoxifen [22].

Taken together, these data introduced the concept that the non-genomic estrogen pathway, in addition to the presence of activating *PIK3CA* mutations, could affect the response to PI3K inhibitors associated with endocrine treatments.

In this study, we first evaluated ER/Src and ER/PI3K interactions in a large cohort of BC patients. We then treated different PDX models of *PIK3CA* mutated and WT breast cancers with the PI3K inhibitor BYL719 combined to fulvestrant and explored their effect on tumor growth as well as on both genomic and non-genomic ER pathways.

## Materials and methods

### Human breast cancer sample collection

The tumors from 440 patients of the Centre Lon Brard (CLB) with invasive non-metastatic BC, whose clinical and biological data were available from the regularly updated institutional database, were analyzed. Written informed consent was obtained from each patient. The study protocol was approved by the institutional ethics committee. Patient characteristics are presented in the additional material (Additional file2, Table S1). In our study, tumors exhibiting less than 10% of ER-positive cells were considered to be ER-negative tumors.

### Patient-derived xenografts

Before PDX establishment, all patients had previously given their verbal informed consent for experimental research on residual tumor tissue available after histopathological analyses. PDX establishment was performed after the approval of the ethics committee of the Institut Curie. According to the French rules and the ethics committee of the Institut Curie, a written consent from patients to obtain residual tumor tissues is not required.

Nine breast cancer PDX models were used in this study. They were established from surgical specimens by grafting tumor fragments into the interscapular fat pad of nude mice as previously described [23, 24]. Female Swiss nude mice, 10 weeks old, were purchased from Charles River (Les Arbresles, France) and maintained under specific pathogen-free conditions. Their characteristics are described in the additional material (Additional file3, Table S2). Their care and housing were in accordance with the institutional guidelines and the rules of the French Ethics Committee (project authorization no. 02163.02). Histological and IHC statuses (ER, PR, and HER2) were determined for the PDXs and compared with that of the patient tumor samples, as described elsewhere [23].

When tumors reached a volume of 60 to 200 mm^3^, mice were randomly assigned to the control or treatment groups, each group consisting of seven or eight mice. Fulvestrant (Faslodex, AstraZeneca, Macclesfield, UK) was administered by intramuscular injection at a dose of 200 mg/kg once a week. BYL719 was purchased from Medchemexpress and was administered orally at 35 mg/kg 5 times per week. Tumor growth was evaluated by measuring two perpendicular diameters of tumors with a caliper twice a week. Individual tumor volumes were calculated as *V* = *a* *b*^2^/2, *a* being the largest diameter and *b* the smallest. Tumor growth inhibition (TGI) of treated tumors versus controls was calculated as the ratio of the mean tumor volume in the treated group to the mean tumor volume in the control group at the same time (end of the experiment). Statistical significance of TGI was calculated using the Mann-Whitney test by comparing the tumor volumes in the treated and control groups. Percent change in tumor volume was calculated for each tumor using the following formula: [(VfV0)/V0]100, where V0 is the initial volume (at the beginning of treatment) and Vf is the final volume (at the end of treatment). Classification of tumor response in waterfall plots: tumor regression, stabilization, and progression corresponded to a percent of volume change lower, equal or > 0, respectively.

Tumor sampling was performed 24 h after the last treatment. No specific toxicity was reported in the experiments; neither diarrhea nor rash was observed, and treated mice did not display any important weight loss throughout the experiment time course.

### Antibodies

**Table:** 

Antibodies	Supplier	Origin	Dilution for PLA	Dilution for IHC
PI3K p85 ab-22653	Abcam	mouse	1/30	
c-Src (B12) sc-8056	SCBT	mouse	1/150	
ER (HC20) sc-542	SCBT	rabbit	1/75	
ER (SP1) 05278406001	Roche	rabbit		Ready to use
p-AKT (Ser473) 4060	CST	rabbit		1/75
p-S6 riboprotein (Ser235/236) 4857	CST	rabbit		1/100
PTEN 9559	CST	rabbit		1/100
PI3K p85 05-212	Millipore	mouse		1/200

### Proximity ligation assay in tissues

This technology, first published in 2006 [20], enables the in situ visualization of protein-protein interactions and was supplied by Sigma. Paraffin-fixed tumor tissues incorporated in TMA blocks were initially sectioned and incubated in a hydrogen peroxide solution, for 5 min at room temperature, to avoid peroxidase quenching. The antibody labeling steps were similar to those described above. For antibody detection, the probes were labeled with horseradish peroxidase after two washes in high purity water. A nuclear staining solution was added to the slides and incubated 2 min at room temperature. After washing the slides for 10 min under running tap water, the samples were consecutively dehydrated in ethanol and xylene. Samples were mounted in a non-aqueous mounting medium and visualized under a bright-field microscope. The protocol has already been optimized for ER/Src and ER/PI3K interactions [18, 21, 25].

### Image acquisition and analysis

The hybridized fluorescent slides were viewed under a Zeiss Axio Imager M2 microscope. Images of three independent zones on each tumor were acquired under identical conditions at 40 magnification. At least, 500 cells were counted per tumor.

### Statistical analysis

ER/Src and ER/PI3K interaction in invasive breast cancer samples (by bright-field microscopy) was quantified as the mean number of dots (denoting interaction) per cell. For the sake of correlation and survival analyses, a cutoff for interaction was defined at the most discriminative difference in DFS and OS as calculated by Kaplan-Meier estimates. Accordingly, ER/Src interaction was defined as high if the mean number of dots/cell > 10 and low if 10 dots/cell, while ER/PI3K interaction was high if > 9 dots/cell and low if 9 dots/cell. Correlations between the 2 biomarkers ER/Src and ER/PI3K were studied. The Pearsons correlation coefficient was presented with asterisks highlighting its significance (*P < 0.05; **P < 0.01; ***P < 0.001). Associations between categorical variables were studied using Pearsons chi-square test. Overall survival (OS) defined as the time from diagnosis to death or date of last follow-up and disease-free survival (DFS) defined as the time from diagnosis to death or relapse or date of last follow-up (for censored patients) were studied.

Survival curves were estimated by the Kaplan-Meier method and compared between the groups with different interaction levels using the log-rank test.

### RT-QPCR analysis

RNA extraction was performed as previously described [26, 27]. Quantitative values were obtained from the number of the cycle (Ct value) at which the increase in the fluorescent signal associated with the exponential growth of PCR products was initially detected by the laser detector of the ABI Prism 7900 sequence detection system (Perkin-Elmer Applied Biosystems, Foster City, CA), using the PE biosystems analysis software according to the manufacturers manuals.

For gene normalization, we used the human TATA box-binding protein (TBP, GenBank accession no. NM_003194). We used protocols for cDNA synthesis and PCR amplification described in detail elsewhere [28]. The results, expressed as *N*-fold differences in target gene expression relative to the TBP gene and termed Ntarget, were determined as Ntarget = 2^Ctsample^, where the Ct value of the sample is obtained by subtracting the average Ct value of the target gene from the average Ct value of TBP gene.

### IHC experiments

Xenografted tumors were fixed in 10% neutral buffered formalin, paraffin embedded, and hematoxylin-eosin-saffron (HES) stained. Outgrowths were analyzed by immunohistochemistry (IHC) for the expression of biomarkers. Immunostaining was performed on a Discovery XT Platform (Ventana Medical System, Tucson, AZ, part of Roche Diagnostics) with antigen retrieval using either EDTA buffer, pH 8.0 (CC1, Ventana Medical System) or citrate buffer 10 mM, pH 6.0 (CC2, Ventana Medical System). Primary antibodies were mostly monoclonal rabbit antibodies, and paired slides immunostained with rabbit IgG were used as negative controls. Incubation and color development involved anti-rabbit multimer secondary antibody (horseradish peroxidase complex) with DAB (3,30-diaminobenzidine tetrahydrochloride) as a substrate (ChromoMap Kit with Anti-rabbit OmniMap, Ventana Medical System). The IHC slides were scanned using a Pannoramic SCAN II (3DHISTECH). We then used the HALO software (Indica Labs) to quantify the expression levels of ER, pAkt (S473), and p-S6riboprotein (S235/6).

## Results

### Clinicopathological characteristics of the patient cohort

Among the 440 patients, 433 had complete clinical data, 430 were assessable for ER/Src interaction, and 417 were assessable for ER/PI3K interaction. The median age at diagnosis was 57.9 years (range 30.4 to 87.4 years). Regarding the tumor stage, 41.8% of patients had tumors beyond 20 mm, and 57.5% displayed axillary LN metastasis. Only 18.9% of patients had SBR grade I tumors, 47.8% had grade II tumors, and 33.3% grade III tumors. ER was positive in 87.1%, PR in 74.8%, and HER2 was overexpressed in 7.2% of the cohort. Table S1 shows the clinico-pathological characteristics of the tested patient cohort (433 patients).

**Fig. 1:**
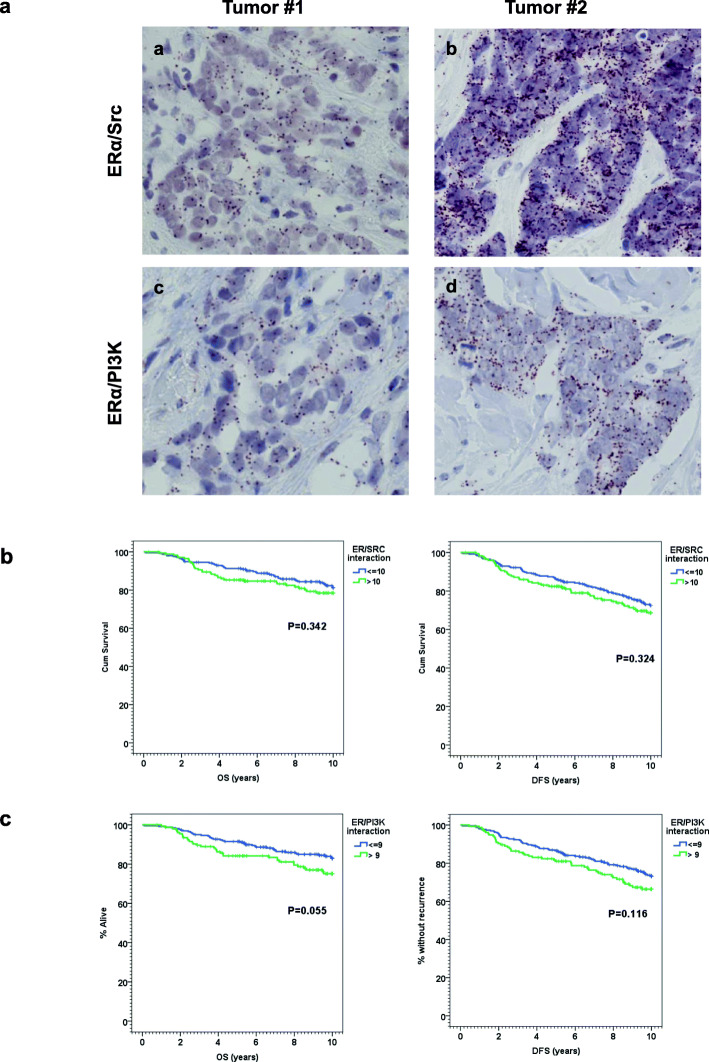
ER/Src and ER/PI3K interactions in human tumoral breast samples. **a** ER/Src (a, b) and ER/PI3K (c, d) interactions were detected by proximity ligation assay (PLA) on two formalin-fixed paraffin-embedded breast tumor sections. The experiments were performed on two serial sections from the same tumor (Obj 40). **b** Kaplan-Meier estimates of OS and DFS according to ER/Src interaction. **c** Kaplan-Meier estimates of OS and DFS according to the ER/PI3K interaction

Representative micrographs of tumor cells with high (tumor#2) and low levels of interaction (tumor#1) of ER/Src and ER/PI3K are shown in Fig.1a. ER/Src interaction was high (> 10 dots) in 174 cases (40.5%), while 256 of cases (59.5%) showed low levels of interaction ( 10 dots). ER/PI3K interaction was high (> 9 dots) in 156 cases (37.4%), while 261 of cases (62.6%) displayed low levels of interaction ( 9 dots). Interestingly, we observed a positive association between ER/Src and ER/PI3K interactions (*P* < 0.001) (Table1). We observed no correlation between high levels of interaction of either ER/Src (Table2) or ER/PI3K (Table3) with any of the traditional prognostic parameters of breast cancer.

**Table 1:** Correlation between ER/Src and ER/PI3K interactions

Variable		ER/PI3K 9, no. (%)	ER/PI3K >9, no. (%)	*P*
ER/Src	Low ( 10)	181 (69.6)	68 (44.2)	**<0.001**
	High (> 10)	79 (30.4)	86 (55.8)

**Table 2:** Distribution of clinical parameters according to ER/Src expression

Variable		ER/SRC 10, no. (%)	ER/SRC >10, no. (%)	P*
Age groups	50 years	76 (29.7)	35 (20.1)	**0.026**
	>50 years	180 (70.3)	139 (79.9)	
T. size	2 cm	152 (59.4)	97 (55.7)	0.455
	>2 cm	104 (40.6)	77 (44.3)	
LN invasion	No	108 (42.2)	74 (42.5)	0.944
	Yes	148 (57.8)	100 (57.5)	
SBR grade	Gr 1	44 (17.2)	37 (21.3)	0.394
	Gr 2	129 (50.4)	77 (44.3)	
	Gr 3	83 (32.4)	60 (34.5)	
ER status	Negative	34 (13.3)	21 (12.1)	0.712
	Positive	222 (86.7)	153 (87.9)	
PR status	Negative	61 (23.8)	47 (27.0)	0.455
	Positive	195 (76.2)	127 (73.0)	
HER2 status	Negative	238 (83.7)	157 (91.3)	0.345
	Positive	16 (6.3)	15 (8.7)	
Breast Cancer subtype	Luminal A	146 (57.0)	95 (54.6)	0.876
	Luminal B	76 (29.7)	58 (33.3)	
	HER2 rich	7 (2.7)	4 (2.3)	
	TNBC	27 (10.5)	17 (9.8)	
Type of adjuvant hormonal	Tamoxifen	93 (42.3)	78 (52.3)	0.057
	AI	127 (57.7)	71 (47.7)

**Table 3:** Distribution of clinical parameters according to the ER/PI3K expression

Variable		ER/PI3K 9, no. (%)	ER/PI3K >9, no. (%)	*P*
Age groups	<50 years	75 (28.7)	36 (23.1)	0.206
	>50 years	186 (71.3)	120 (76.9)	
T. size	<2 cm	157 (60.2)	84 (53.8)	0.207
	>2 cm	104 (39.8)	72 (46.2)	
LN invasion	No	115 (44.1)	60 (38.5)	0.262
	Yes	146 (55.9)	96 (61.5)	
SBR grade	Gr 1	54 (20.7)	25 (16.0)	0.069
	Gr 2	130 (49.8)	68 (43.6)	
	Gr 3	77 (29.5)	63 (40.4)	
ER status	Negative	31 (11.9)	22 (14.1)	0.509
	Positive	230 (88.1)	134 (85.9)	
PR status	Negative	60 (23.0)	45 (28.8)	0.182
	Positive	201 (77.0)	111 (71.2)	
HER2 status	Negative	238 (93.0)	144 (92.3)	0.802
	Positive	18 (7.0)	12 (7.7)	
Breast Cancer subtype	Luminal A	155 (59.4)	77 (49.4)	0.249
	Luminal B	75 (28.7)	57 (36.5)	
	HER2 rich	6 (2.3)	5 (3.2)	
	TNBC	25 (9.6)	17 (10.9)	
Type of adjuvant hormonal	Tamoxifen	97 (57.1)	65 (49.2)	0.151
	AI	129 (42.9)	67 (50.8)	

### High levels of ER/PI3K interaction are associated with poorer breast cancer patient outcome

No significant impact on either OS (HR=1.24; 95% CI 0.791.94; *P*=0.343) or DFS (HR=1.21; 95% CI 0.831.75; *P*=0.325) was noted for patients displaying high or low levels of ER/Src interactions (Fig.1b). Conversely, ER/PI3K interaction predicted a trend towards poorer OS and DFS (Fig.1c), with an 8-year OS rate of 79.2% in patients with low levels versus 72.4% in patients with high levels of ER/PI3K interaction (HR = 1.55; 95% CI 0.992.44; *P* = 0.055) and an 8-year DFS rate of 79.2% in patients with low levels versus 72.4% in patients with high levels of ER/PI3K interaction (HR = 1.35; 95% CI 0.931.97; *P* = 0.116).

### Targeting estrogen genomic and non-genomic signaling in ER-positive PDX models

Based on the present data and our previous results [21, 22], we hypothesized that the estrogen non-genomic pathway could represent a therapeutic target in BC and particularly in endocrine-resistant ER+ BCs. To test our hypothesis, we targeted non-genomic signaling using a combination of endocrine therapy (fulvestrant), known to degrade ER, inhibiting transcription of its target genes, and a PI3K inhibitor, as our team formerly showed that inhibiting PI3K activity disrupted the complex containing ER/PI3K and its downstream signaling in MCF-7 cells [21]. As our previous results were obtained with LY294002, an inhibitor not used in clinics, we studied the effect of three other PI3K inhibitors on MCF-7 cells and found that BYL719 was the most effective at decreasing the interaction of ER with PI3K (Additional file4, Fig. S1). This inhibitor was thus selected for further in vivo experiments. We hence evaluated the efficacy of fulvestrant alone, BYL719 alone, or BYL719 + fulvestrant (combination) in 6 PDX models of ER+ breast cancers. The characteristics of the different PDXs are summarized in the Additional file3 Table S2 and Additional file5 Fig. S2. Five of these models were established from primary breast tumors and one from bone metastasis. Three models (HBCx-86, HBCx-91, and BC1111) are *PIK3CA* mutated (HBCx-86: p.E545K HBCX-91 and BC1111: p.H1047R).

#### The hormone-sensitive ER+ *PIK3CA* WT HBCx-34 PDX model

**Fig. 2:**
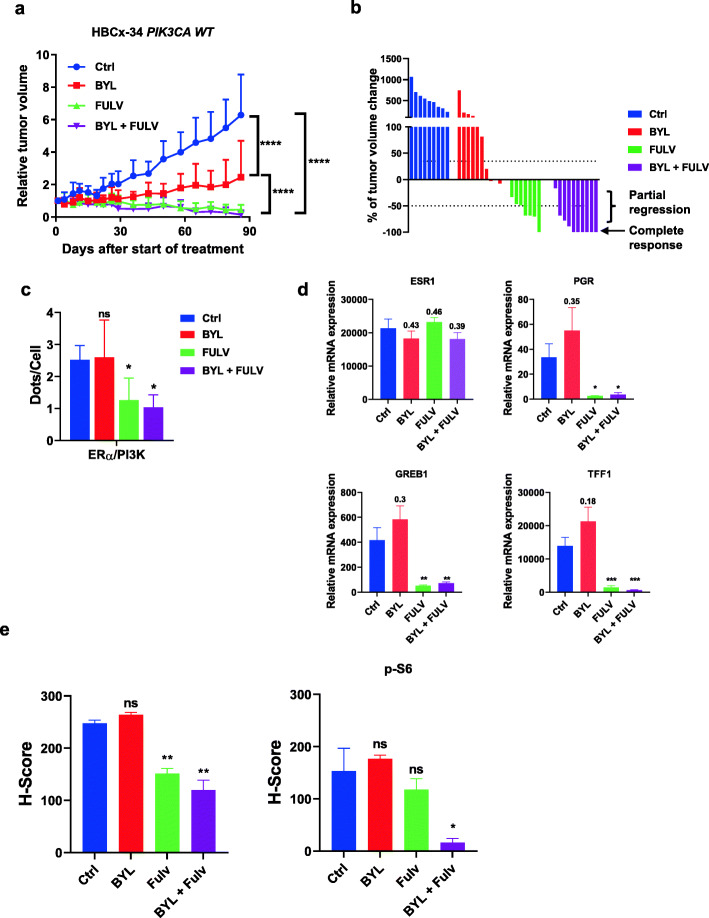
In vivo drug response to BYL719 or fulvestrant alone and combined in the HBCx-34 PDX model. **a** Effect of the different treatments on HBCx-34 tumor growth. Each treatment included 10 mice; the *y*-axis indicates the mean of RTV + SD. **b** Waterfall plot representing the percent of change in tumor volume from baseline in individual HBCx-34 xenografts in the different treatment groups. **c** PLA was performed on treated tumors embedded in paraffin to study the interactions between ER and PI3K. Quantification was performed by counting the number of signals per cell in five independent zones of the section (*n* > 500 cells counted/tumor). Significance (*P* value) between treatments was determined using the Student *t* test. ns, non-significant; *P < 0.05; **P < 0.01. **d** Expression of estrogen-regulated genes (ERG) analyzed by RT-QPCR in PDX tumor samples (*N*=4). **e** IHC staining was performed on formalin-fixed paraffin-embedded PDX tumors using anti-ER and anti-P-S6 riboprotein (S235/6) antibodies. Quantification of highly, medium, and negative cells was performed as described in the Materials and methods section. Significance (*P* value) between treatments and controls were performed using the *t* test. ns:not significant

In this model, treatment with fulvestrant for 3 months resulted in tumor regression in 5/8 xenografts, stable disease in 1 xenograft, and complete response in 1 xenograft, though BYL719 alone had only a mild but significant effect in comparison (Fig.2a). Interestingly, tumor response further increased in the combination group (*P* = 0.01, Mann-Whitney test) with 6/10 xenografts displaying complete responses, 3 tumor regression, and 1 stable disease (Fig.2b).

At the level of ER non-genomic signaling, the ER/PI3K interaction was significantly reduced by fulvestrant alone or when combined to BYL719, though BYL719 alone had no effect (Fig.2c). The analysis of some estrogen-regulated genes (ERG) showed a non-significant increase in *PGR*, *GREB1*, and *TFF1* gene expression in BYL719-treated xenografts and a significant decrease in the expression of the same genes in xenografts treated with fulvestrant or the combination (Fig.2d). *ESR1* expression remained unchanged. IHC staining validated that ER expression decreased upon fulvestrant treatment and that BYL719 inhibited downstream PI3K signaling only when combined with fulvestrant, as evidenced by P-S6 riboprotein (S235/6) expression (Fig.2e, Additional file6 Fig. S3). However, as this tumor does not express P-Akt (S473), we were unable to confirm the efficacy of BYL719 on PI3K signaling.

#### The hormone-sensitive ER+ *PIK3CA* WT HBCx-3 and *PIK3CA* mut HBCx-86 PDX models

**Fig. 3:**
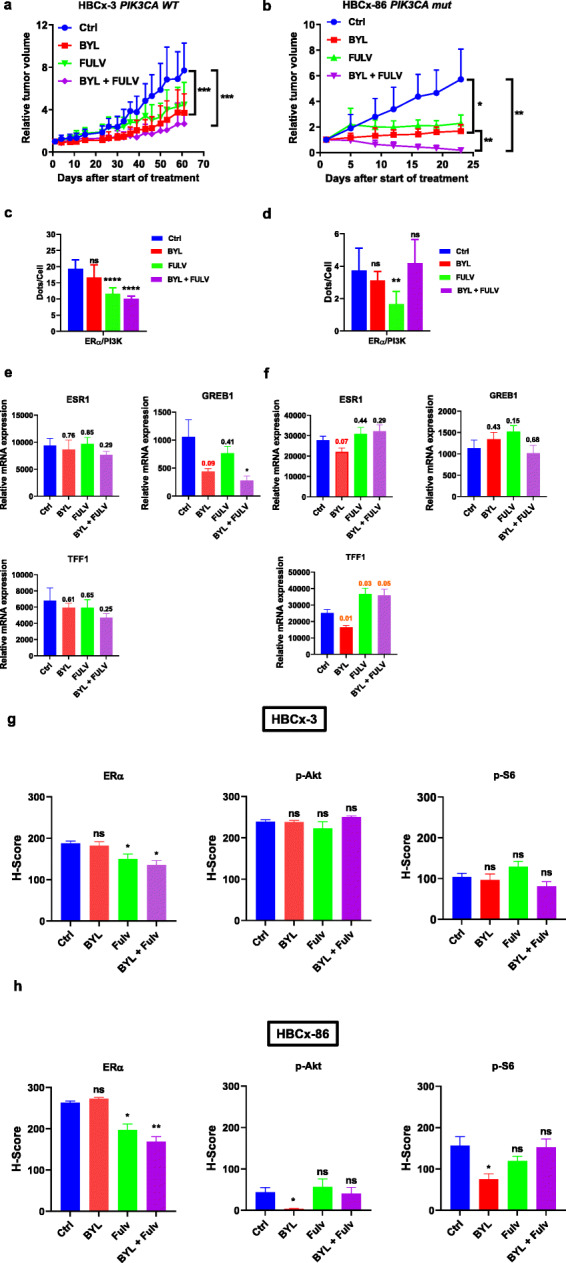
In vivo drug response to BYL719 or fulvestrant alone and combined in the HBCx-3 and HBCx-86 PDX models. **a** Effect of the different treatments on HBCx-3 tumor growth. Each treatment included 10 mice; the *y*-axis indicates the mean of RTV + SD. **b** Effect of the different treatments on HBCx-86 tumor growth. **c**, **d** PLA was performed and analyzed as in Fig.2. ns, non-significant; *P < 0.5; **P < 0.01. **e**, **f** RT-QPCR was performed from RNA extracted from frozen tumor samples using specific primers for ERG. **g**, **h** IHC staining was performed on formalin-fixed paraffin-embedded PDX tumors using anti-ER, anti-P-AKT (S473), and anti-P-S6 riboprotein (S235/6) antibodies. Quantification of highly, medium, and negative cells was performed as described in the Materials and methods section. Significance (*P* value) between treatments and controls were performed using the *t* test. ns: not significant

These two models responded partially to BYL719 and fulvestrant alone, whereas their combination clearly increased this anti-tumoral effect. In the PDX HBCx-3, the combination of BYL719 and fulvestrant did not completely inhibit tumor growth (Fig.3a), although this was significantly decreased compared to the control (TGI of 62% and 65%, respectively). The *PIK3CA-*mutated HBCx-86 model responded to the combination by exhibiting remarkable tumor regression (Fig.3b). For these models, ER/PI3K interaction was efficiently disrupted with fulvestrant but not with BYL719 alone (Fig.3c, d). The combination strongly decreased ER/PI3K interaction in the HBCx-3 model, whereas it had no effect on the HBCx-86 model. ERG remained largely unaffected by the treatment (Fig.3e, f). IHC staining revealed that for both models, fulvestrant treatment decreased ER expression (Fig.3g, h, Additional file7, Fig S4, Additional file8 Fig S5). However, regarding BYL719 efficacy, it had no effect on the HBCx-3 model (Fig.3g, Additional file7 Fig. S4), whereas it significantly decreased P-S6 riboprotein (S235/6) staining in HBCx-86 (Fig.3h, Additional file8 Fig S5). Unfortunately, we could not confirm this result, as Akt staining was too low. These results suggest that the effects of fulvestrant on tumor growth are potentiated following PI3K inhibition in the context of estrogen non-genomic signaling.

#### The fulvestrant-resistant ER+ *PIK3CA* WT TamR HBCx-22 and *PIK3CA* mut BC1111 models

**Fig. 4:**
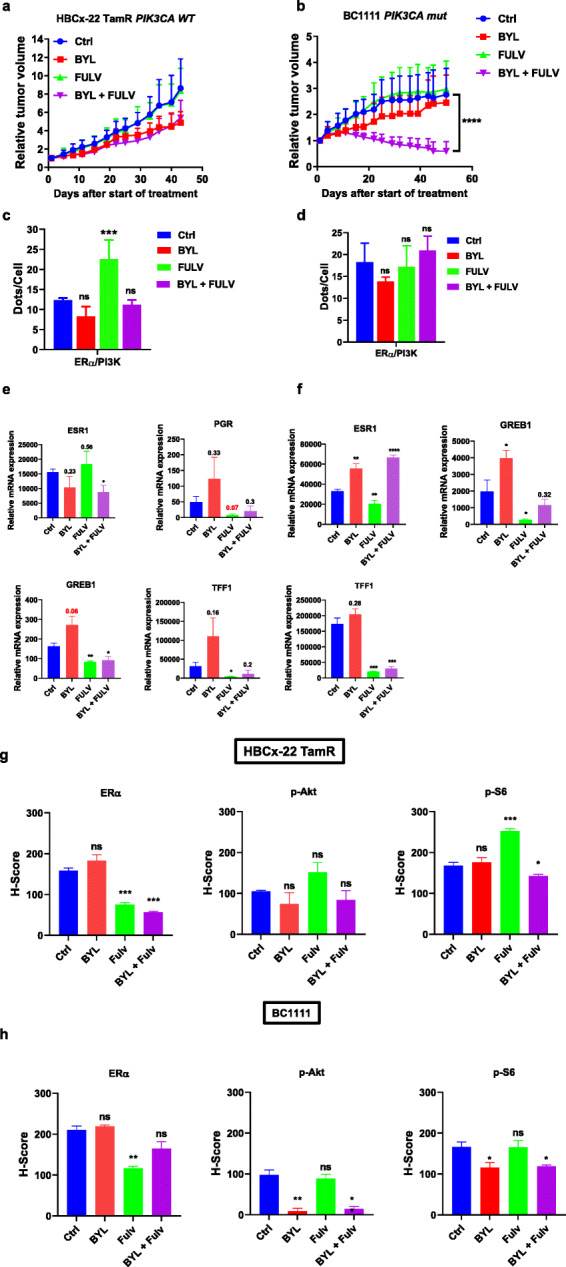
In vivo drug response to BYL719 or fulvestrant alone and combined in the HBCx-22 TamR and BC1111 PDX models. **a** Effect of the different treatments on HBCx-22 TamR tumor growth. Each treatment included 10 mice; the *y*-axis indicates the mean of RTV + SD. **b** Effect of the different treatments on HBCx953 tumor growth**. c**, **d** PLA was performed and analyzed as in Fig.2. **e**, **f** RT-QPCR was performed from RNA extracted from frozen tumor samples using specific primers for ERG. **g**, **h** IHC staining was performed on formalin-fixed paraffin-embedded PDX tumors using anti-ER, anti-P-AKT (S473), and anti-P-S6 riboprotein (S235/6) antibodies. Quantification of highly, medium, and negative cells was performed as described in the Materials and methods section. Significance (*P* value) between treatments and controls were performed using the *t* test. ns: not significant

The HBCx-22 TamR model did not respond significantly to BYL719 alone or in combination with fulvestrant (Fig.4a). Surprisingly, the BC1111 model was resistant to BYL719, but the combination strongly inhibited tumor growth (TGI 79%, *P* < 0.0001) (Fig.4b). Interestingly, in the two models, ER/PI3K interaction was not significantly affected by the treatment, fulvestrant having an opposite effect in the HBCx-22 TamR model by significantly increasing this interaction (Fig.4c, d), corroborating our previous findings [22]. The expression of ERG diminished following the administration of fulvestrant or of the combination treatment, whereas it increased with BYL719 alone (Fig.4e, f). IHC staining of the HBCx-22 TamR model revealed that fulvestrant strongly inhibited ER nuclear expression, while BYL719 had no effect on PI3K signaling (Fig.4g, Additional file9 Fig S6). With regard to the BC1111 model, fulvestrant triggered a decrease in ER expression, while BYL719 efficiently inhibited the PI3K pathway (Fig.4h, Additional file10 Fig S7).

#### The fulvestrant-resistant ER+ *PIK3CA* mut HBCx-91 model

**Fig. 5:**
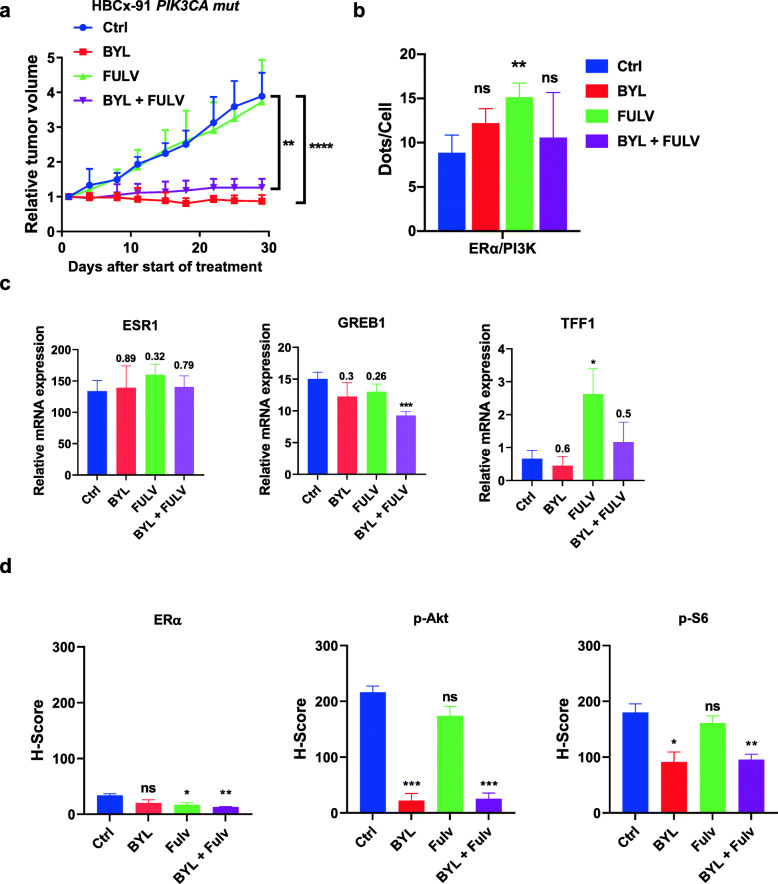
In vivo drug response to BYL719 or fulvestrant alone and combined in the HBCx-91 PDX model**. a** Effect of the different treatments on HBCx-91 tumor growth. Each treatment included 10 mice; the *y*-axis indicates the mean of RTV + SD. **b** PLA was performed and analyzed as in Fig.2. **c** RT-QPCR was performed from frozen tumor for ERG expression. **d** IHC staining was performed on formalin-fixed paraffin-embedded PDX tumors using anti-ER, anti-P-AKT (S473), and anti-P-S6 riboprotein (S235/6) antibodies. Quantification of highly, medium, and negative cells was performed as described in the Materials and methods section. Significance (*P* value) between treatments and controls were performed using the *t* test. ns: not significant

This PDX model was engrafted with a tumor from a patient expressing a low level of ER and harboring a *PI3KCA* mutation. This model was resistant to fulvestrant alone but responded to BYL719 alone or in combination with fulvestrant, by inducing a stable low-grade disease (Fig.5a). We observed a significant increase in ER/PI3K interaction upon fulvestrant treatment, whereas BYL719 alone or in combination had no effect (Fig.5b). The expression of ERG was not significantly affected by the different treatments (Fig.5c). The IHC staining confirmed that ER was faintly expressed in the nucleus of tumoral cells (Fig.5d). Fulvestrant induced a significant decrease in ER expression and BYL719 efficiently targeted the PI3K pathway (Fig.5d, Additional file11 Fig S8).

### Targeting estrogen non-genomic signaling in ER-negative PDX models

As the estrogen non-genomic complex is also activated in ER-negative breast tumors [21] and the PI3K pathway is active in TNBC, we tested the combination of BYL719 + fulvestrant in 3 ER-negative PDX models.

**Fig. 6:**
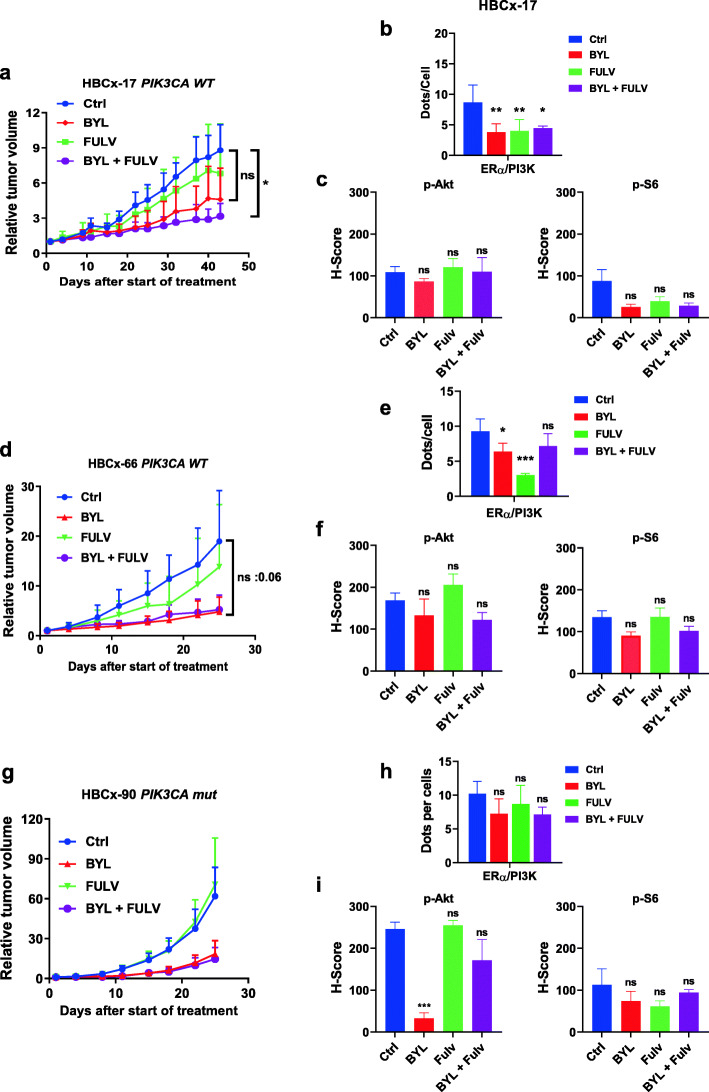
In vivo drug response to fulvestrant, BYL719 alone, and combined in 3 ER-negative models. **a** Effect of the different treatments on HBCx27 tumor growth. Each treatment included 10 mice; the *y*-axis indicates the mean of RTV + SD. **b** PLA was performed and analyzed as in Fig.2. **c** IHC staining was performed on formalin-fixed paraffin-embedded PDX tumors using anti-P-S6 riboprotein (S235/6) antibody. **d** Effect of the different treatments on HBCx-801 tumor growth. Each treatment included 10 mice; the *y*-axis indicates the mean of RTV + SD. **e** PLA was performed and analyzed as in Fig.2. **f** IHC staining was performed on formalin-fixed paraffin-embedded PDX tumors using anti-P-AKT (S473) and anti-P-S6 riboprotein (S235/6) antibodies. **g** Effect of the different treatments on HBCx-90 tumor growth. Each treatment included 10 mice; the *y*-axis indicates the mean of RTV + SD. **h** PLA was performed and analyzed as in Fig.2. **i** IHC staining was performed on fixed PDX tumors using anti-P-AKT (S473) and anti-P-S6 riboprotein (S235/6) antibodies. Quantification of highly, medium, and negative cells was performed as described in the Materials and methods section. Significance (*P* value) between treatments and controls were performed using the *t* test.ns: not significant

In the HBCx-17 model (WT for *PIK3CA*), only the combination of BYL719 + fulvestrant inhibited tumor growth with a TGI of 64% (*P* = 0.03, Mann-Whitney *t* test), although no tumor regression was observed (Fig.6a). Interestingly, fulvestrant and BYL719 alone significantly decreased ER/PI3K formation, whereas the combination had no cumulative effect (Fig.6b). IHC analysis revealed a similar decrease in P-S6 riboprotein (S235/236) expression in tumors treated with BYL719 alone or combined with fulvestrant, although it was less clear for P-Akt (Fig.6c, Additional file11 Fig S9).

In the HBCx-66 model (WT for *PI3KCA*), fulvestrant had a modest effect on tumor growth, while administration of BYL719 alone or in combination led to a strong decrease in tumor volume (Fig.6d). BYL719 and fulvestrant significantly decreased ER/PI3K interaction whereas the combination had no significant effect (Fig.6e). Similarly to the previous ER model, BYL719 showed a non-significant decrease in P-AKT (S473) and P-S6 riboprotein (S235/236) staining (Fig.6f, Additional file12 Fig S10).

In the HBCx-90 PDX (*PI3KCA* mutated), treatment with fulvestrant had no effect on tumor growth, whereas BYL719 or the combination significantly decreased tumor volume. Interestingly, in this model resistant to fulvestrant, the anti-estrogen had no effect on ER/PI3K interactions (Fig.6h). Conversely, BYL719 significantly inhibited the downstream PI3K pathway but did not affect ER/PI3K interaction (Fig.6i, Additional file13 Fig S11).

In conclusion, in ER-negative tumors, the effect of fulvestrant on tumor growth is likely linked to its ability to disrupt ER interaction with PI3K.

## Discussion

Based on our results and other existing literature, we postulated that the components of estrogen non-genomic signaling could constitute both new prognostic markers and new therapeutic targets. In this study, we sought to validate the activation of this pathway in aggressive breast cancers in a new cohort of breast tumor patients. ER/Src/PI3K being a hallmark of non-genomic signaling, we studied ER/Src and ER/PI3K by in situ PLA in samples of 440 invasive breast tumors. Interestingly, we found that their high level of expression was correlated with a trend to poorer patient survival, ER/PI3K being associated with the most pronounced effects. These data corroborate those obtained in the first cohort of 175 BCs [21] and argue in favor of targeting ER/PI3K in in vivo models of BCs.

As a proof-of-concept, we decided to target ER/PI3K interactions using an anti-estrogen (fulvestrant) or a PI3K inhibitor alone (BYL719) or in combination in 6 models of ER+ and 3 ER BC PDXs. For the ER-positive models, we evaluated their effect on tumor growth as well as on estrogen non-genomic signaling (by studying ER/PI3K interaction) and on genomic signaling (by studying the expression of ER target genes). For the ER-negative models, we assessed the efficacy of treatments on tumor growth and on ER/PI3K interactions. We decided to use a PI3K inhibitor acting predominantly against PI3K, as it has been largely shown by our team and others that treating BC cells with PI3K inhibitors disrupts ER/PI3K interactions in ER-positive cell lines [13, 15, 21]. We confirmed this result in the present study using BYL719 and showed that it was able to disrupt ER/PI3K interactions in MCF-7 cells. We found that BYL719 efficacy on downstream signaling pathways was restricted to PDX mutated for *PIK3CA* as described by Fritsch et al. [29]. Our present work reveals that conversely to the results obtained in cells, BYL719 had no significant effect in vivo on ER/PI3K interactions in ER+ PDX models tested. However, we do not believe that this effect was attributable to in vivo versus in vitro experimental settings (PDX versus cells) as in two ER-negative models (HBCx-17 and HBCx-66), we clearly observed a significant decrease in ER/PI3K interaction upon BYL719 treatment. We can hypothesize that the lack of efficacy could vary according to the breast cancer subtype. Indeed, MCF-7 cells correspond to the luminal A subtype. Unfortunately, all ER-positive PDX models used herein were of a luminal B subtype. This type of cancer may be refractory to the PI3K inhibitor, or at least to its effect at dissociating the ER/PI3K interaction. These results suggest that it would be of interest to find novel molecules able to destabilize this interaction. As a proof of concept, Aurrichios team showed that a peptide targeting the site of interaction between ER/Src was able to disrupt the ER/Src/PI3K complex formation, as well as cell proliferation in vitro and in vivo [30]. We also found that this peptide is able to restore tamoxifen sensitivity in a model of MCF-7 cells resistant to tamoxifen [22].

In summary, of the 6 PDX of ER+ BCs tested, HBCx-34, HBCx-86, HBCx-3, and BC1111 responded to the combination of BYL719+ fulvestrant, and HBCx-86, HBCx-3, and BC1111 were *PIK3CA* mutated. Activation of the non-genomic ER pathway decreased in treated tumors of 3 PDXs, due largely to fulvestrant and was not always associated with the in vivo response (HBCx-3). The combination of BYL719 and fulvestrant was more efficient than fulvestrant alone in 3 models; however, this effect was not associated with decreased levels of ER/PI3K complex in xenografts treated with the combination compared to fulvestrant-treated xenografts. Similarly, PI3K-dependent regulation of ER transcription was observed only in 3 PDXs and was not correlated to *PIK3CA* mutations nor to the response to the PI3K inhibitor. However, in order to obtain a strong tumor response to combined therapy, it is necessary to simultaneously inhibit genomic and non-genomic signaling. Indeed, complete responses were obtained in HBCx-34 xenografts, where both pathways were inhibited. However, when only one pathway was inhibited, the response was partial, as evidenced for HBCx3 and HBCx86, in which only the non-genomic pathway was inhibited by fulvestrant, whereas for HBCx-22 TamR and BC1111 models, only the genomic pathway was inhibited. For the HBCx-91 model, the response was partial and both estrogen signaling pathways remained unresponsive to fulvestrant, probably due to a very low level of ER. Interestingly, in the 3 models resistant to fulvestrant, ER/PI3K was not disrupted. Inversely, in 2 cases, their interaction increased, although ER was efficiently degraded in the nucleus and ERG expression was downregulated. This is in accordance with recent results from our lab showing that ER/PI3K interaction increases upon resistance to endocrine therapy [22]. This could be due to a stabilization of ER by PI3K enzymatic activity. Indeed, PI3K is able to phosphorylate ER on Serine 167 [31], phosphorylation is involved in ER degradation, and PI3K inhibitors have been shown to increase its degradation [32]. Unlike previous findings [12], we observed no increase in ER expression at the mRNA or the protein levels in all ER-positive models treated with BYL719, likely due to the different models investigated (in cellulo vs in vivo).

Concerning ER-negative models, in HBCx-17 and HBCx-66 tumors, fulvestrant had a modest effect on growth inhibition. Interestingly, in these models, fulvestrant alone was able to decrease PI3K pathway signaling probably by disrupting ER/PI3K interactions which might affect PI3K activity and thus downstream signaling. Conversely, in the HBCx-90 model, where fulvestrant had no effect on tumor growth, neither ER/PI3K interaction nor the downstream pathway was inhibited.

Altogether, our results confirm that the ER/PI3K interaction could be evaluated before associating endocrine therapy with PI3K inhibitors in BC. Moreover, targeting this interaction may improve the response to endocrine therapy in ER-positive tumors and patient survival in ER-negative BCs.

## Conclusions

In summary, the present study identifies ER/PI3K interaction, a hallmark of estrogen non-genomic signaling, as a new potential biomarker associated with a decrease in BC patient survival. In addition, targeting this interaction may circumvent resistance to endocrine therapies in ER-positive tumors and could contribute to decreasing tumor growth in ER-negative tumors.

## Supplementary Information

**Additional file 1: Supplementary methods**.

**Additional file 2: Table S1**. Baseline characteristics for the patients included in the TMA study.

**Additional file 3: Table S2**. Characteristics of the PDX models.

**Additional file 4: Figure S1**. Effect of PI3kinase inhibitors on ER/PI3K dimer formation. A. Oestrogen-deprived MCF-7 cells treated or not with PI3K inhibitors LY294002, BYL219, GDC-0032 and GDC-0941 (5 M) 15 min before E2 treatment were incubated with E2 (10-8 M) for 5 min (see Additional supplementary methods). After fixation, in situ PLA for ER/PI3K was performed. The detected dimers are represented by red dots. The nuclei were counterstained with DAPI (blue) (Obj:X63). B. Quantification of the number of dots per cell was performed using image J software and an automated analysis (see Additional supplementary methods). *P* value: *** <0.01, **** < 0.001.

**Additional file 5: Figure S2**. IHC staining was performed on formalin-fixed paraffin-embedded BC1111 PDX tumor using anti-PTEN antibody.

**Additional file 6: Figure S3**. Proximity ligation assay (PLA) was performed on treated HBCx-34 tumors embedded in paraffin to study the interactions between ER and PI3K. IHC staining was performed on the same tumors using anti-ER, P-AKT (S473) and anti-P-S6 riboprotein (S235/6) antibodies.

**Additional file 7: Figure S4**. Proximity ligation assay (PLA) was performed on treated HBCx-3 tumours embedded in paraffin to study the interactions between ER and PI3K. IHC staining was performed on the same PDX tumours using anti-ER, P-AKT (S473) and anti-P-S6 riboprotein (S235/6) antibodies.

**Additional file 8: Figure S5**. Proximity ligation assay (PLA) was performed on treated HBCx-86 tumours embedded in paraffin to study the interactions between ER and PI3K. IHC staining was performed on the same PDX tumours using anti-ER, P-AKT (S473) and anti-P-S6 riboprotein (S235/6) antibodies.

**Additional file 9: Figure S6**. Proximity ligation assay (PLA) was performed on treated HBCx-22 TamR tumours embedded in paraffin to study the interactions between ER and PI3K. IHC staining was performed on the same PDX tumours using anti-ER, P-AKT (S473) and anti-P-S6 riboprotein (S235/6) antibodies.

**Additional file 10: Figure S7**. Proximity ligation assay (PLA) was performed on treated BC1111 tumors embedded in paraffin to study the interactions between ER and PI3K. IHC staining was performed on the same PDX tumors using anti-ER, P-AKT (S473) and anti-P-S6 riboprotein (S235/6) antibodies.

**Additional file 11: Figure S8**. Proximity ligation assay (PLA) was performed on treated HBCx-91 tumours embedded in paraffin to study the interactions between ER and PI3K. IHC staining was performed on the same PDX tumours using anti-ER, P-AKT (S473) and anti-P-S6 riboprotein (S235/6) antibodies.

**Additional file 12: Figure S9**. A. Proximity ligation assay (PLA) was performed on treated HBCx-17 tumours embedded in paraffin to study the interactions between ER and PI3K. B. IHC staining was performed on the same PDX tumours using anti-ER, P-AKT (S473) and anti-P-S6 riboprotein (S235/6) antibodies.

**Additional file 13: Figure S10**. A. Proximity ligation assay (PLA) was performed on treated HBCx-66 tumours embedded in paraffin to study the interactions between ER and PI3K. B. IHC staining was performed on the same PDX tumours using anti-ER, P-AKT (S473) and anti-P-S6 riboprotein (S235/6) antibodies.

**Additional file 14: Figure S11**. A. Proximity ligation assay (PLA) was performed on treated HBCx-90 tumours embedded in paraffin to study the interactions between ER and PI3K. B. IHC staining was performed on the same PDX tumours using anti-ER, P-AKT (S473) and anti-P-S6 riboprotein (S235/6) antibodies.

**Additional file 15.** Reference list.

## Data Availability

All data in our study are available upon request.
