# Supplementary material for: Analysis of genomic and non-genomic signaling of estrogen receptor in PDX models of breast cancer treated with a combination of the PI3K inhibitor alpelisib (BYL719) and fulvestrant
Source: Breast Cancer Res. 2021 May 21;23:57. doi: 10.1186/s13058-021-01433-8 (PMC8139055; doi:10.1186/s13058-021-01433-8)
Supplement: Supplementary file 1 — Additional file 1: Supplementary methods. [file 13058_2021_1433_MOESM1_ESM.docx]

**Additional file 1: Supplementary Methods**

**Cell culture**

MCF-7 cells from the ATCC were maintained at 37°C in Dulbecco’s Modified Eagle’s Medium (DMEM) supplemented with 10% fetal calf serum and 1% non-essential amino acids. Prior to experiments, the cells were grown 48 hr in phenol red-free DMEM and 10% charcoal-treated serum (Biowest), then stimulated 5 min with E_2_ (Sigma). When stated, MCF-7 cells were treated with PI3K inhibitors LY294002 (Millipore), BYL219 (Sellekchem), GDC-0032 (Synkinase) and GDC-0941 (Synkinase).

**Proximity Ligation assay on fixed cells.**

This technology exposes protein/protein interactions *in situ* [1]. Briefly, cells were seeded and fixed with cold methanol. After saturation, the different couples of primary antibodies were incubated for 1 hr at 37°C. The PLA probes consisting of secondary antibodies conjugated with complementary oligonucleotides were incubated for 1 hr at 37°C. The amplification step followed the ligation of nucleotides for 100 min at 37°C. Samples were subsequently analysed under fluorescence microscopy.

**Image acquisition and analysis**

The hybridized fluorescent slides were viewed under a Nikon Eclipse Ni microscope. Images were acquired under identical conditions at x60 magnification. Image acquisition was performed by imaging DAPI staining at a fixed Z Position while a Z stack of +/- 5 μm at 1 μm intervals was carried out. The final images were stacked to a single level before further quantification. On each sample, at least 100 cells were counted. Analyses and quantification of these samples were performed using the Image J software following our published method [2]. This software includes a ‘‘Counter cells’’ plugin, which enables users to count and analyse the number of cells/dots present on an 8-bit colour image.
