# Supplementary material for: Analysis of genomic and non-genomic signaling of estrogen receptor in PDX models of breast cancer treated with a combination of the PI3K inhibitor alpelisib (BYL719) and fulvestrant
Source: Breast Cancer Res. 2021 May 21;23:57. doi: 10.1186/s13058-021-01433-8 (PMC8139055; doi:10.1186/s13058-021-01433-8)
Supplement: Supplementary file 3 — Additional file 3: Table S2. Characteristics of the PDX models. [file 13058_2021_1433_MOESM3_ESM.docx]

**Table S2: Characteristics of the PDX models**

| PDX | Origin | IHC | PIK3CA status | PTEN  status | Reference |
| --- | --- | --- | --- | --- | --- |
| HBCx-3 | Primary tumour | ERα+ PR- | Wild-type | - | [3] |
| HBCx-22 TamR | Primary tumour | ERα+ PR+ | Wild-type | - | [4] |
| HBCx-34 | Primary tumour | ERα+ PR+ | Wild-type | + | [4] |
| HBCx-86 | Primary tumour | ERα+ PR- | p.E545K | + | [5] |
| HBCx-91 | Primary tumour | ERα+ PR- | p.H1047R | + | [5] |
| BC1111 | Bone metastasis | ERα+ PR- | p.H1047R | + | Figure S2 |
| HBCx-17 | Primary tumour | Triple-negative | Wild-type | - | [5] |
| HBCx-66 | Primary tumour | Triple-negative | Wild-type | - | [5] |
| HBCx-90 | Primary tumour | Triple-negative | p.H1047R | - | [5] |
