# Supplementary material for: Analysis of genomic and non-genomic signaling of estrogen receptor in PDX models of breast cancer treated with a combination of the PI3K inhibitor alpelisib (BYL719) and fulvestrant
Source: Breast Cancer Res. 2021 May 21;23:57. doi: 10.1186/s13058-021-01433-8 (PMC8139055; doi:10.1186/s13058-021-01433-8)
Supplement: Supplementary file 4 — Additional file 4: Figure S1. Effect of PI3kinase inhibitors on ER/PI3K dimer formation. A. Oestrogen-deprived MCF-7 cells treated or not with PI3K inhibitors LY294002, BYL219, GDC-0032 and GDC-0941 (5 M) 15 min before E2 treatment were incubated with E2 (10-8 M) for 5 min (see Additional supplementary methods). After fixation, in situ PLA for ER/PI3K was performed. The detected dimers are represented by red dots. The nuclei were counterstained with DAPI (blue) (Obj:X63). B. Quantification of the number of dots per cell was performed using image J software and an automated analysis (see Additional supplementary methods). P value: *** <0.01, **** < 0.001. [file 13058_2021_1433_MOESM4_ESM.docx]

**
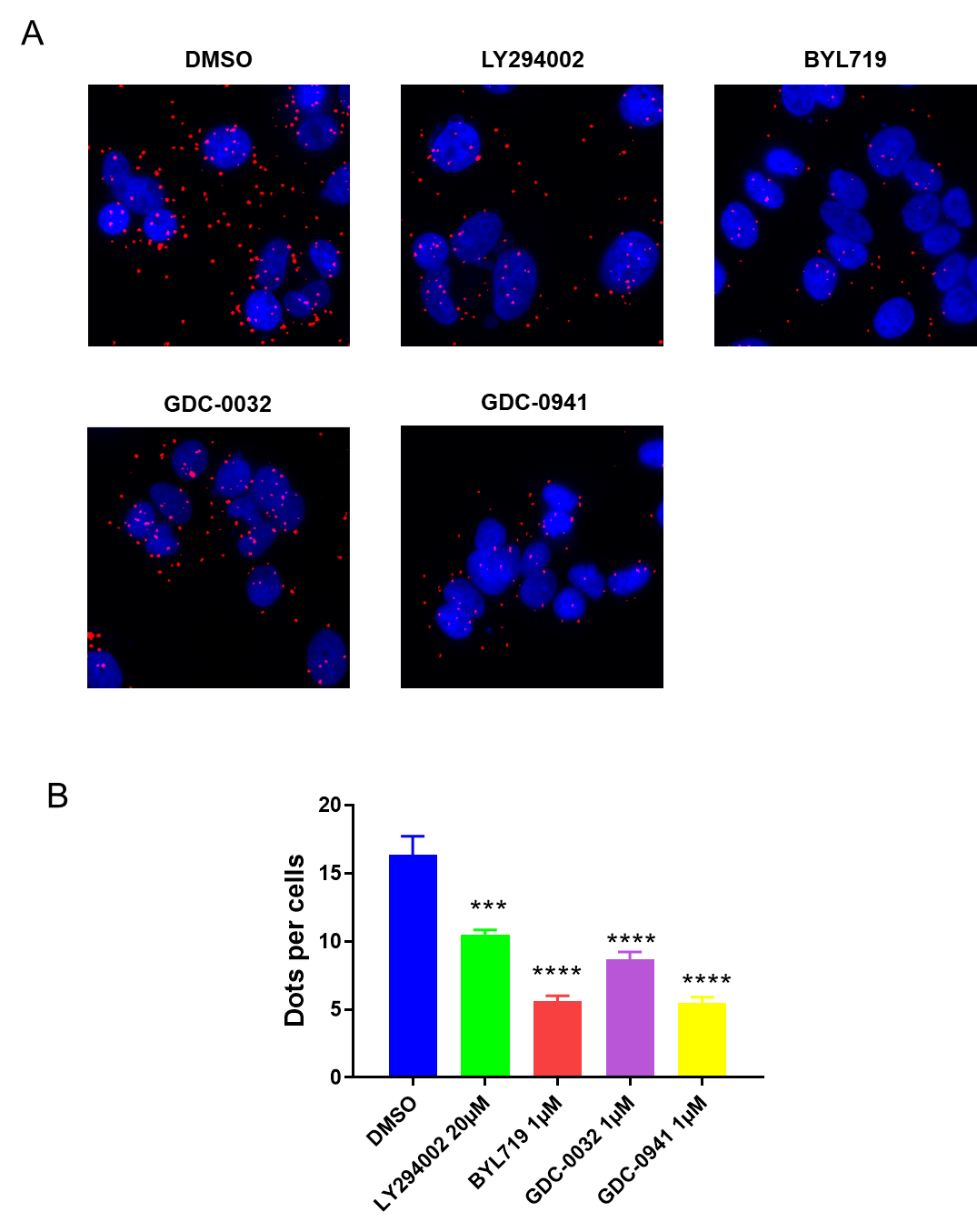
**

**Figure S1.** Effect of PI3kinase inhibitors on ERα/PI3K dimer formation**.** **A**. Oestrogen-deprived MCF-7 cells treated or not with PI3K inhibitors LY294002, BYL219, GDC-0032 and GDC-0941 (5 µM) 15 min before E_2_ treatment were incubated with E_2_ (10^-8^ M) for 5 min (see Additional supplementary methods). After fixation, *in situ* PLA for ERα/PI3K was performed. The detected dimers are represented by red dots. The nuclei were counterstained with DAPI (blue) (Obj:X63). **B**. Quantification of the number of dots per cell was performed using image J software and an automated analysis (see Additional supplementary methods). ). P-value: *** <0.01, **** < 0.001
