# Supplementary material for: Analysis of genomic and non-genomic signaling of estrogen receptor in PDX models of breast cancer treated with a combination of the PI3K inhibitor alpelisib (BYL719) and fulvestrant
Source: Breast Cancer Res. 2021 May 21;23:57. doi: 10.1186/s13058-021-01433-8 (PMC8139055; doi:10.1186/s13058-021-01433-8)
Supplement: Supplementary file 5 — Additional file 5: Figure S2. IHC staining was performed on formalin-fixed paraffin-embedded BC1111 PDX tumor using anti-PTEN antibody. [file 13058_2021_1433_MOESM5_ESM.docx]

**
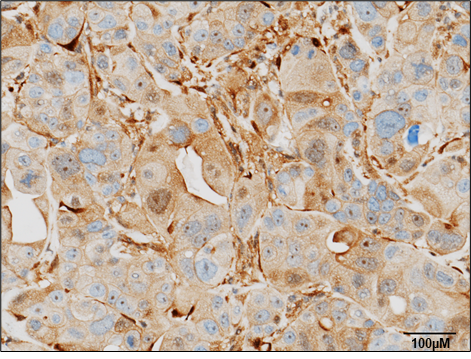
**

**Figure S2.** IHC staining was performed on formalin-fixed paraffin-embedded BC1111 PDX tumor using anti-PTEN antibody.
