# Supplementary material for: Analysis of genomic and non-genomic signaling of estrogen receptor in PDX models of breast cancer treated with a combination of the PI3K inhibitor alpelisib (BYL719) and fulvestrant
Source: Breast Cancer Res. 2021 May 21;23:57. doi: 10.1186/s13058-021-01433-8 (PMC8139055; doi:10.1186/s13058-021-01433-8)
Supplement: Supplementary file 6 — Additional file 6: Figure S3. Proximity ligation assay (PLA) was performed on treated HBCx-34 tumors embedded in paraffin to study the interactions between ER and PI3K. IHC staining was performed on the same tumors using anti-ER, P-AKT (S473) and anti-P-S6 riboprotein (S235/6) antibodies. [file 13058_2021_1433_MOESM6_ESM.docx]

**
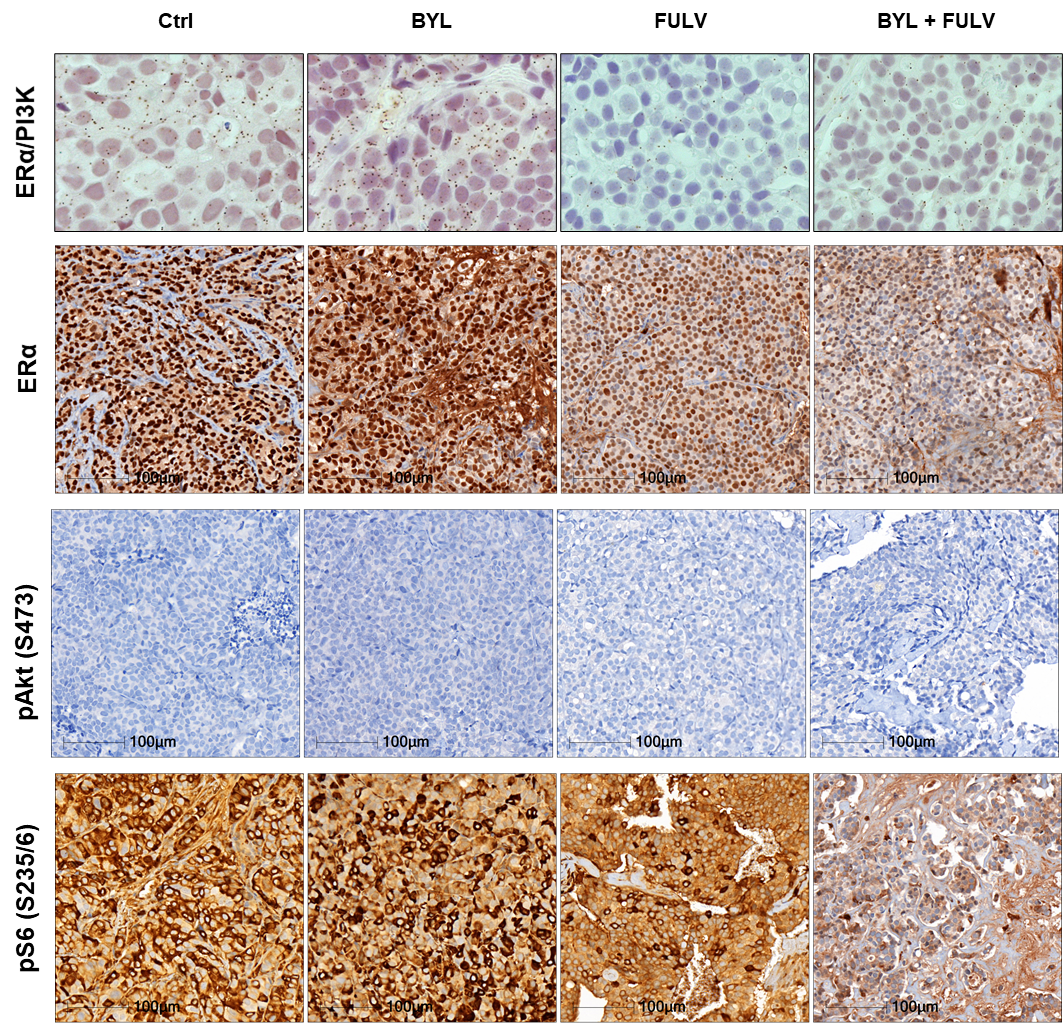
**

**Figure S3:** Proximity ligation assay (PLA) was performed on treated HBCx-34 tumors embedded in paraffin to study the interactions between ERα and PI3K. IHC staining was performed on the same tumors using anti-ERα, P-AKT (S473) and anti-P-S6 riboprotein (S235/6) antibodies.
