# Supplementary material for: Analysis of genomic and non-genomic signaling of estrogen receptor in PDX models of breast cancer treated with a combination of the PI3K inhibitor alpelisib (BYL719) and fulvestrant
Source: Breast Cancer Res. 2021 May 21;23:57. doi: 10.1186/s13058-021-01433-8 (PMC8139055; doi:10.1186/s13058-021-01433-8)
Supplement: Supplementary file 10 — Additional file 10: Figure S7. Proximity ligation assay (PLA) was performed on treated BC1111 tumors embedded in paraffin to study the interactions between ER and PI3K. IHC staining was performed on the same PDX tumors using anti-ER, P-AKT (S473) and anti-P-S6 riboprotein (S235/6) antibodies. [file 13058_2021_1433_MOESM10_ESM.docx]

**
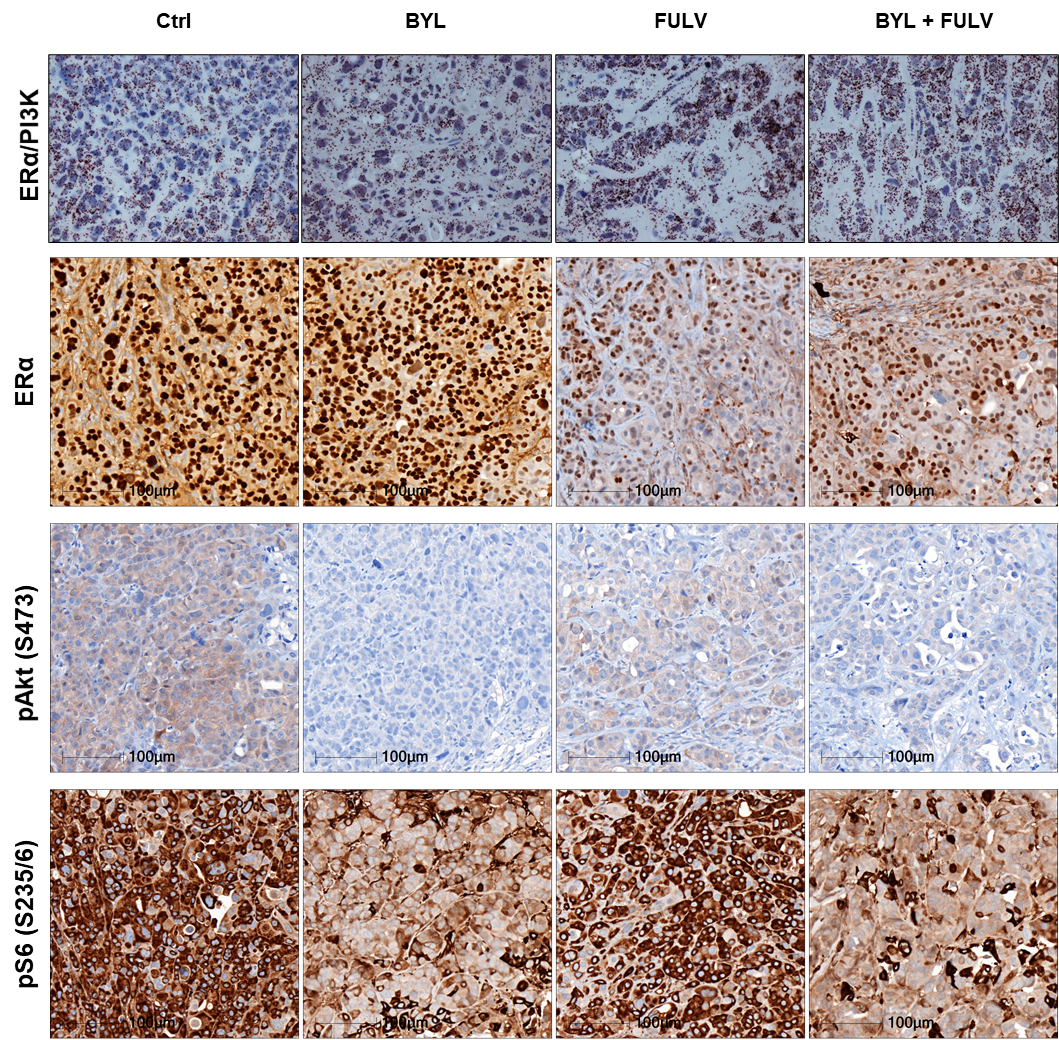
**

**Figure S7:** Proximity ligation assay (PLA) was performed on treated BC1111 tumors embedded in paraffin to study the interactions between ERα and PI3K. IHC staining was performed on the same PDX tumors using anti-ERα, P-AKT (S473) and anti-P-S6 riboprotein (S235/6) antibodies.
