# Supplementary material for: Analysis of genomic and non-genomic signaling of estrogen receptor in PDX models of breast cancer treated with a combination of the PI3K inhibitor alpelisib (BYL719) and fulvestrant
Source: Breast Cancer Res. 2021 May 21;23:57. doi: 10.1186/s13058-021-01433-8 (PMC8139055; doi:10.1186/s13058-021-01433-8)
Supplement: Supplementary file 15 — Additional file 15. Reference list. [file 13058_2021_1433_MOESM15_ESM.docx]

Reference List

1. Soderberg O, Gullberg M, Jarvius M, Ridderstrale K, Leuchowius KJ, Jarvius J, et al (2006)

Direct observation of individual endogenous protein complexes in situ by proximity ligation.

Nat. Methods 3: 995-1000.

2. Poulard C, Jacquemetton J, Trédan O, Cohen P, Treilleux I, Marangoni E and Le Romancer M (2019) Oestrogen non-genomic signalling is activated in tamoxifen-resistant breast cancer. IJMS, PMID 31195751.

3. Cottu P, Marangoni E, Assayag F, de CP, Vincent-Salomon A, Guyader C, de PL, Elbaz C, Karboul N, Fontaine JJ, Chateau-Joubert S, Boudou-Rouquette P, Alran S, Dangles-Marie V, Gentien D, Poupon MF, Decaudin D (2012) Modeling of response to endocrine therapy in a panel of human luminal breast cancer xenografts. Breast Cancer Res Treat 133: 595-606. 10.1007/s10549-011-1815-5 [doi].

4. Cottu P, Bieche I, Assayag F, El BR, Chateau-Joubert S, Thuleau A, Bagarre T, Albaud B, Rapinat A, Gentien D, de la Grange P, Sibut V, Vacher S, Hatem R, Servely JL, Fontaine JJ, Decaudin D, Pierga JY, Roman-Roman S, Marangoni E (2014) Acquired resistance to endocrine treatments is associated with tumor-specific molecular changes in patient-derived luminal breast cancer xenografts. Clin Cancer Res 20: 4314-4325. 1078-0432.CCR-13-3230 [pii];10.1158/1078-0432.CCR-13-3230 [doi].

5. Coussy F, de KL, Lavigne M, Bernard V, Ouine B, Boulai A, El BR, Dahmani A, Montaudon E, Assayag F, Morisset L, Huguet L, Sourd L, Painsec P, Callens C, Chateau-Joubert S, Servely JL, Larcher T, Reyes C, Girard E, Pierron G, Laurent C, Vacher S, Baulande S, Melaabi S, Vincent-Salomon A, Gentien D, Dieras V, Bieche I, Marangoni E (2019) A large collection of integrated genomically characterized patient-derived xenografts highlighting the heterogeneity of triple-negative breast cancer. Int J Cancer 145: 1902-1912. 10.1002/ijc.32266 [doi].
